# Event and Comment

**Published:** 1916-11

**Authors:** 


### DENTAL REGISTER

Vol. LXX NOVEMBER, 1916 No. 11

### EVENT AND COMMENT

PASSING OF THE PIONEERS. We chronicle on another page of this issue the close of life for four men who contributed, each in his own peculiar way, to the development of the dental profession in a very definite and commendable manner. Every man who has taken the slightest interest in professional affairs either knew personally or had heard of Dr. George L. Field of Detroit, and Drs. Loomis P. Haskell and C. R. E. Koch of Chicago, and the older workers in the profession will know of Dr. A. T. Metcalf of Battle Creek, Mich. These men would have been men of mark in any calling, but, fortunately, they worked in dentistry, and humanity has and will bless them and all such for their life-long devotion to so noble a cause. All four of these men were strong mentally and physically, and would have graced any calling, and possibly might have become more famous or wealthy in some other calling, and we may think they perhaps are not entitled to our encomiums. We shall perhaps get a truer conception of the moral and spiritual characteristics of such men if we turn our attention to the history and development of our profession associated with their professional careers. It is often difficult to say whether it is men that make history or events in the lives of men that develops men of character and distinction. When men espouse a cause with all their powers, there can be little question but the cause is benefited, but it may not always be clear that the personality is developed relatively. It also seems as though the more difficult the task, and the more self-denial demanded, the greater the development of the spiritual and heroic in character. These men lived and labored in the constructive period of our professional existence and became important factors in this remarkable development; in fact, it is hard to disassociate them. We sometimes wonder if any big men are developing in our profession today. There is not perhaps the same necessity for extraordinary men today, and yet no one can believe that we do not need big men today. Is it not a fact that our present development makes such large and intricate demands that of necessity it has been subdivided and parceled out to a larger number of men, and, consequently, instead of a few brilliant stars, we have a large number of brilliant men who are jostling one another, with the result that our stars are obscured in the dust of the crowd? It may be that we have so many heroes today that we fail to recognize them.

Both Dr. Field and Dr. Metcalf had done their active work and retired from practice for several years, but their careers and even their personalities will long remain as pleasant memories to the older men in the profession of Michigan. Early in their careers they contended strenuously for ethical standards, they labored incessantly for higher technical and educational accomplishments, and they lived to see their beloved profession respected and even honored by people of culture and refinement and taking its place as one of the great benevolent callings of the entire world. Whether or not they were ever conscious of their influence in this development is immaterial and was perhaps of no consequence to them; but the lives of all such men should be honored by the living, and they should be a source of inspiration to the young men now entering the profession. Dr. Haskell was probably the oldest practicing dentist in the country and known to every dentist for his work and writings.

The pioneering is as yet not all done; in fact, the profession is just awaking to the possibilities of our calling. Preventive medicine is the slogan of today. No one knows how it is to come. We have exhausted our resources almost in our attempts to battle successfully with the spread of disease, and there seems but one way left for immediate investigation. The next great step is up to the dental profession, we are told. It will not be taken by our predecessors, but it will come by the same untiring devotion and zeal which characterized their careers, and which now compel us to stop and review their lives with admiration. Shall the dead live again?

UNITED STATES NAVY DENTAL CORPS. We are printing in this issue the announcement of the call for dental surgeons in the Navy. This is a splendid opportunity for a few of our young men to engage in the grand work of caring for the health of our country's defenders. Graduates of dental schools, not over thirty years old, may take up this service, providing they are citizens of this country. All candidates must be physically sound and must pass an examination as to their educational and technical training. Dental officers have a definite rank and pay, the same as other officers. The examinations for admission are held in Washington, D. C., at frequent intervals, which can be learned by addressing an inquiry to Dr. W. C. Braisted, Surgeon General United States Navy.

This opens up a new and useful avenue of dental influence, which it is hoped may get the confidence and interest of the best men graduating from our dental schools. It is an exacting work for which only the best graduates are qualified, and for the good name of the dental profession we trust all the vacancies may soon be filled by bright and energetic young men.

